# The neoantigen and associated tumour microenvironment in long-term survivors with oesophageal adenocarcinoma

**DOI:** 10.3389/fimmu.2026.1926114

**Published:** 2026-09-21

**Authors:** James M. Lonie, Sandra Brosda, Venkateswar Addala, Bijun Zeng, Rituparna Bhatt, Vanessa F. Bonazzi, Lauren G. Aoude, Lambros T. Koufariotis, Kalpana Patel, Katina D. Hulme, Clemence J. Belle, Scott Wood, John V. Pearson, Nicola Waddell, Riccardo Dolcetti, Andrew P. Barbour

**Affiliations:** 1 Frazer Institute, The University of Queensland, Brisbane, QLD, Australia; 2 Department of Upper Gastrointestinal Surgery, Princess Alexandra Hospital, Brisbane, QLD, Australia; 3 Medical Genomics, QIMR Berghofer Medical Research Institute, Brisbane, QLD, Australia; 4 Faculty of Health, Medicine and Behavioural Sciences, The University of Queensland, Brisbane, QLD, Australia; 5 Peter MacCallum Cancer Centre, Melbourne, VIC, Australia; 6 Sir Peter MacCallum Department of Oncology, The University of Melbourne, Melbourne, VIC, Australia; 7 Immunopathology Group, Mater Research Institute, The University of Queensland, Brisbane, QLD, Australia; 8 Department of Microbiology and Immunology, The University of Melbourne, Melbourne, VIC, Australia

**Keywords:** genomics, immunology, neoantigens, oesophageal adenocarcinoma, tumour microenvironment

## Abstract

**Background:**

Oesophageal adenocarcinoma (OAC) is a highly mutated cancer with poor survival. While outcomes are improving, long-term survival remains elusive for the majority of patients. Immunotherapy in combination with chemotherapy has shown benefit but is effective in only a subset of patients, highlighting the lack of reliable genomic biomarkers for response and survival. Effective anti-tumour immune responses, including those targeting neoantigens, are critical for durable outcomes. However, their role in long-term OAC survivors remains poorly understood.

**Methods:**

Whole-genome, whole-exome, and RNA sequencing data was used to identify (expressed) putative neoantigens from two independent OAC cohorts. We combined data from the Extended-DOCTOR (ExtDOC; n=149) and the TCGA ESCA (n=88) cohort to profile the neoantigen landscape in OAC. 95 patients of the ExtDOC cohort were enrolled in the DOCTOR trial, sponsored by the GI Cancer Trials. Selected putative neoantigens from 14 patients underwent *in vitro* validation using HLA-matched healthy donor and patient-derived PBMCs.

**Results:**

We identified a median of 23 neoantigens per tumour sample which were mostly 9- and 10-mers predicted to bind to HLA-A molecules. Low stromal and immune exclusion scores as well as high-quality neoantigens with high expressed differential agretopicity index (DAI), a measure of immunogenicity, were linked to good survival outcomes. We observed intra-tumour heterogeneity and found immunogenic putative neoantigens for 12/14 patients. The tumour environment (TME) revealed three immune clusters which were associated with patient outcomes.

**Conclusions:**

We showed that OAC is characterised by a complex and largely immunosuppressive TME. In our cohort, patients with an immune-enriched TME had more favourable outcomes, including higher response rates, lower disease recurrence and longer survival. This might be linked to the presence of high-quality neoantigens. Together, these findings provide a rationale for the development of tailored, immune-informed treatment strategies in OAC.

## 1 Introduction

For patients with curable oesophageal adenocarcinoma (OAC), current standard of care treatment is empiric, with poor survival outcomes. Treatment involves perioperative chemotherapy or neoadjuvant chemoradiotherapy followed by surgery +/- (neo)adjuvant immunotherapy (1, 2). For advanced/metastatic patients, chemotherapy remains the backbone of treatment but can include targeted therapy or immunotherapy (1, 2). Although recent immunotherapy trials have shown promise, response to treatment remains limited to a small subset of patients (3, 4). While the molecular structure of OAC has been studied, a thorough understanding of the neoantigen landscape and tumour microenvironment (TME) is just beginning to emerge (5, 6). Given the genomic complexity and highly heterogenous nature of OAC (7, 8), developing a comprehensive understanding of the immune microenvironment is challenging, but necessary to improve clinicopathological outcomes.

The basic tenet of immune responses in cancer revolves around genetic mutations (9). These mutations can lead to the formation of immunogenic tumour neoantigens, which, when presented on the tumour cell surface, can induce adaptive immune responses (10, 11). Neoantigens are associated with immunotherapy response and survival (12). The ability of cancers to generate immunogenic neoantigens has been seen in both highly immunogenic cancers such as melanoma (13), and cancers with immune excluded/desert phenotypes and low mutation rates such as pancreatic ductal adenocarcinoma (14). Personalised neoantigen vaccines have shown promise in multiple solid cancers (15–17). To date, the role of neoantigens in OAC remains largely unexplored, with few studies describing their landscape and associated immune responses (8, 18, 19). Mutagenic tumours with higher neoantigen loads show increased CD8+ T cell infiltration (19), but limited studies have assessed *in vitro* immunogenicity, with one study identifying immunogenic neoantigens in approximately 10% of cases (18).

We performed comprehensive *in silico* and *in vitro* characterisation of the neoantigen landscape and TME in OAC, including patients from the GI Cancer Trials DOCTOR clinical phase II trial (ACTRN12609000665235) (20). The aim of this study was to define associations with clinical outcomes and assess neoantigen immunogenicity in long-term survivors. These findings provide a foundation to inform future neoantigen-based therapeutic strategies.

## 2 Methods

### 2.1 Patient cohorts and sample collection

We analysed treatment-naïve primary tumour and blood samples from 149 OAC patients from The Cancer Evolution Biobank and GI Cancer Trials DOCTOR trial (ACTRN12609000665235) (20) (**Table 1**). Overall survival (OS) was defined from first consultation to death or last follow-up and major pathological response (MPR) to neoadjuvant therapy (NAT) as <10% residual tumour cells with p/ypN0 stage (21).

**Table 1:** Sample and patient characteristics by cohort.

Cohort	ExtDOC	TCGA	*In-vitro* validation
Patient number	149	88	14
DNAseq samples	117 (78.5%)	88 (100%)	14 (100%)
WES	27 (23.1%)	88 (100%)	10 (71.4%)
WGS	90 (76.9%)	0	4 (28.6%)
RNAseq samples	115 (77.2%)	82 (93.2.8%)	14 (100%)
Median Age at diagnosis (range)	63 (38–83)	70 (27–86)	59 (48–70)
DOCTOR trial patient	95 (63.8%)	0	4 (28.6%)
Sex			
Male	134 (89.9%)	76 (86.4%)	1 (7.1%)
Female	15 (10.1%)	12 (13.6%)	13 (92.9%)
Clinical stage (cTNM)			
0	1 (0.7%)	0	0
I	4 (2.7%)	2 (2.2%)	2 (14.3%)
II	26 (17.4%)	4 (4.6%)	3 (21.4%)
III	95 (63.8%)	8 (9.1%)	6 (42.9%)
IVA	14 (9.4%)	4 (4.6%)	2 (14.3%)
IVB	7 (4.7%)	2 (2.2%)	1 (7.1%)
N/A | Not reported	2 (1.3%)	68 (77.3%)	0
Treatment			
Neoadjuvant CRT	48 (32.2%)	6 (6.8%)	5 (35.7%)
Neoadjuvant CT	70 (47%)	4 (4.5%)	5 (35.7%)
Direct to Surgery	16 (10.7%)	73 (83%)	1 (7.1%)
Palliative	12 (8.1%)	5 (5.7%)	1 (7.1%)
Endoscopic management	2 (1.3%)	0	2 (14.3%)
Definitive CRT	1 (0.7%)	0	0
Pathological stage ([y]pTNM)			
I	44 (29.5%)	11 (12.5%)	6 (42.9%)
II	23 (15.4%)	24 (27.3%)	3 (21.4%)
III	44 (29.5%)	36 (40.9%)	1 (7.1%)
IVA	13 (8.7%)	3 (3.4%)	1 (7.1%)
IVB	2 (1.3%)	0	0
N/A | Not reported	23 (15.4%)	14 (15.9%)	3 (21.4%)
Major pathological response			
Yes	27 (18.1%)	0	4 (28.6%)
No	92 (61.7%)	0	6 (42.9%)
N/A | Not reported	30 (20.1%)	88 (100%)	4 (28.6%)
Pathological nodal status			
N0	68 (45.6%)	0	9 (64.3%)
N1+	59 (39.6%)	0	2 (14.3%)
N/A | Not reported	22 (14.8%)	88 (100%)	3 (21.4%)
Disease recurrence/progression			
Yes	82 (55%)	0	5 (35.7%)
No	66 (44.3%)	0	9 (64.3%)
N/A | Not reported	1 (0.7%)	88 (100%)	0

Values represent median (range), or frequency (%). CRT, chemoradiotherapy; CT, chemotherapy; N/A, not applicable; RNA-seq, RNA sequencing; WES, whole-exome sequencing; WGS, whole-genome sequencing.

Tumour biopsies were stored in RNAlater^®^ (ThermoFisher Scientific, USA). Peripheral whole blood was collected for matched normal DNA and peripheral blood mononuclear cell (PBMC) isolation before and after NAT where possible. Healthy donor blood was obtained via the Australian Red Cross. PBMCs were isolated from 10 mL whole blood using Ficoll-Paque (Sigma-Aldrich, USA) and density centrifugation with phosphate-buffered saline (PBS) overlay. After centrifugation, the PBMC layer was collected, washed twice, counted, and cryopreserved or used immediately.

DNA and RNA were extracted from tissue using AllPrep DNA/RNA mini kits (Qiagen, Germany) and buffy coat DNA using Flexigene kits (Qiagen, Germany). Whole-exome sequencing (WES) and RNA-seq BAM files for 88 OAC patients were obtained from the TCGA ESCA cohort (https://portal.gdc.cancer.gov/projects/TCGA-ESCA).

### 2.2 DNA-sequencing

Paired-end WES on a HiSeq2000 (Illumina, USA) was performed on matching tumour and buffy coat DNA from 27 patients after library preparation (SureSelect V7-Post). Paired-end whole-genome sequencing (WGS) on a HiSeq XTen (Illumina, USA) was performed for 90 patients after library preparation (TruSeq PCR free Library kit). Sequencing depth was 30x for normal (buffy coat) and 100x (WES) or 60x (WGS) for tumour samples. Average read length was 150 bp.

Following adaptor trimming (Cutadapt v1.9) (22), reads were aligned to GRCh37 (BWA-MEM v0.7.15, SAMtools) (23), duplicated reads marked (Picard MarkDuplicates v2.8.15) (24), and variants called (qSNP v2.1.4, GATK HaplotypeCaller v4.0.4.0) (25, 26) and annotated (SnpEff v2.1.2) (27). Tumour purity was estimated using ascatNGS (WGS) (28) and Sequenza (WES) (29).

### 2.3 RNA-sequencing and analyses

115 tumour samples underwent library preparation (TruSeq Stranded messenger RNA (mRNA)) and sequencing with 100 bp paired-end reads (Macrogen, South Korea). After trimming (Cutadapt v1.9) (30), reads were aligned to GRCh37 (STAR v2.5.2a) (31) and gene expression estimated (RSEM v1.2.30) (32). Transcripts-per-million data and published gene sets (33, 34) were used for cell-type enrichment (GSVA) (35). Hierarchical unsupervised clustering was performed using Euclidean distance with Ward D2 linkage and permutation (*n* = 1000) (ComplexHeatmap v3.19) (36). Tumour immune dysfunction and exclusion (TIDE) scores were calculated (http://tide.dfci.harvard.edu) (37).

### 2.4 HLA typing and neoantigen prediction

Class I HLA genotypes were determined from tumour-normal WGS/WES (Optitype v1.3.1 and Polysolver) (38, 39). Variants were annotated (VEP v86) (40). Neoantigens were predicted (pVAC-Seq v4.0.10, NetMHCpan v4.0) (41, 42), retaining epitopes with binding affinity inhibitory concentration (IC50) ≤500 nM (<50 nM as strong binders) (43). Expression was confirmed by RNA-seq (≥10 mutant reads; qbasepileup) (44) and the differential agretopicity index (DAI) was calculated for each neoantigen (43, 45).

In patients with available PBMCs, the criteria used to select putative neoantigens for *in vitro* immunogenicity assessment included: predicted HLA class I binding (with a preference for predicted binding to HLA-A*02), confirmed tumour mRNA expression, and predicted binding affinity (IC50 <500 nM). These criteria were used to identify putative neoantigens with the greatest likelihood of being expressed, presented by tumour cells, and recognised by adaptive immune cells. Where multiple candidate neoantigens were identified, peptides with the highest DAI values were selected for functional validation assays. Peptides were synthesised to >95% purity (GL Biochem, China).

### 2.5 Monocyte-derived dendritic cells and *in vitro* expansion of neoantigen-specific T cells

Monocyte-derived dendritic cells (MoDCs) were generated from healthy donor PBMCs using plastic adherence. Adherent cells were cultured with interleukin (IL)-4 (Peprotech^®^, USA) and GM-CSF (Peprotech^®^) to produce immature dendritic cells, refreshed on day 3, and matured with lipopolysaccharide (Invitrogen™, USA) on day 5 before harvest.

Autologous lymphocytes were co-cultured with MoDCs (10:1) and stimulated with peptide (2 µg/mL). Positive (CEF: HLA-restricted influenza epitope) and negative controls (mock: cRPMI + 0.5% DMSO) were included. Cytokines (IL-2, IL-7, and IL-15) were added and refreshed every 2–3 days, with repeat peptide stimulation on day 9. After 14 days, antigen-specific expanded T cells were harvested. Each biological replicate had at least 3 technical replicates.

### 2.6 Cytotoxicity assay

Expanded antigen-specific T cells (effector cells) were co-cultured with T2A2 (antigen-specific killing) or K562 cells (non-antigen-specific killing) at 20:1, 10:1, and 5:1 ratios.

Target cells were stained with CellTrace™ Violet (CTV; Invitrogen™; CTV^high^ for peptide-pulsed, CTV^low^ for non-peptide-pulsed), pulsed with individual target epitopes where applicable, mixed at 1:1, and incubated with effector cells for 5 hours. Target-only controls measured spontaneous cell death. Cells were washed and stained for viability with Viability Stain (AF700, 1:8000, BD Biosciences, USA).

### 2.7 Detection of neoantigen-specific T cell responses

PBMCs were cultured for 6 hours with neoepitopes (10 µg/mL), CEF peptides (2 µg/mL peptide), or mock control, in the presence of CD107a-AF488 (BD Biosciences), GolgiPlug™ (brefeldin A); and GolgiStop™ (monensin). Cells were washed, stained for viability with Live/Dead Fixable Aqua™ (Invitrogen™) overnight, then surface stained for T cell markers (CD3-BUV395, CD4-BUV737, CD8-BV785, CD45RA-PE-Cy7, and CCR7-PerCP-Cy5.5), fixed, and permeabilised using Cytofix/Cytoperm™ (BD Biosciences). Intra-cellular staining was performed for IFNγ-BV421 (BD Biosciences), IL-2-BV650 (BD Biosciences), and TNFα-PE (BD Biosciences) before final washing and flow cytometry.

### 2.8 Flow cytometry and antigen-specific cytotoxic lysis

Experiments were performed on an LSR Fortessa-X20 flow cytometer (BD Biosciences, USA), with PMT voltages standardised based on unstained cell viability. Data was analysed in FlowJo (v≥10.2; Tree Star, USA).

Epitope-specific lysis was assessed by gating live, single CTV-stained cells and comparing of CTV^high^ and CTV^low^ populations. Percent antigen-specific lysis was calculated relative to controls. Peptides were tested in 2 (non-HLA-A2 binding) or 3 (HLA-A2 binding) healthy donors and considered immunogenic if lysis was observed in at least one (**Supplementary Table 1**).

### 2.9 Statistical analysis

Statistical analyses were performed in R (version ≥ 3.5.3) (46) using a significance level of 0.05 and Benjamini-Hochberg false discovery rate (FDR) correction. Fisher’s exact and Wilcoxon rank-sum test were used for categorical and continuous variables, respectively, Spearman correlation for correlation analyses, and survival analyses used Kaplan-Meier, log-rank, and Cox models (survival v3.4.0, survminer v0.4.9) (47–49).

## 3 Results

### 3.1 Patient cohort

We profiled the neoantigen and immune landscape of 237 OAC patients. We combined patients from the TCGA ESCA cohort (n=88) and the Extended-DOCTOR (ExtDOC) cohort (n=149), including 95 DOCTOR trial patients (**Table 1**). 205 patients (86.6%) had DNA-sequencing with either WES (n=115) or WGS (n=90). RNA-seq data was available for 197 patients (82.8%), of whom 155 patients (65.1%) had matched DNA and RNA-seq.

The median age of disease onset was 63 (range 38-83) in the ExtDOC cohort, and 69.5 (range 27-86) in the TCGA cohort (**Table 1**). Most ExtDOC patients received neoadjuvant therapy (NAT), with 47% receiving chemotherapy (CT), and 32% receiving chemoradiotherapy (CRT), while most TCGA patients proceeded directly to surgery (83%). The ExtDOC cohort showed significantly longer overall survival (OS) than the TCGA cohort (median OS 51 months vs. 23 months, p<0.001; **Supplementary Figure S1**), as treatment with NAT results in significantly improved survival (50, 51).

### 3.2 Mutation and neoantigen landscape in OAC

We focused on protein coding regions to profile the mutational and putative neoantigen landscape using WES and WGS data (n=205), given neoantigens can derive from any coding mutation (**Supplementary Figure S2A**). A higher TMB was observed in WES compared to WGS samples (median 5.3 vs 6.58, p<0.01, **Supplementary Figure S2B**). However, no difference between the putative neoantigen load was observed. TCGA samples had a higher median putative neoantigen load (median 30 vs 18, p<0.05, **Supplementary Figure S2B**; **Supplementary Table 2**). The overall median TMB was 5.67 (range 0.38-70.16; **Figure 1A**) and 6,346 putative neoantigens were predicted with a median of 23 per tumour sample (range 0-265; mean 30.96; **Figure 1B**). Predicted neoantigen load showed positive correlations with TMB (r=0.69, p<0.001; **Figure 1B**) and tumour purity (r=0.39, p<0.001; **Figure 1C**). The majority of neoantigens were 9- and 10-mers (**Figure 1D**) and were mainly derived from missense mutations (91.68%; **Figure 1E**) in commonly mutated cancer genes such as *TP53*, and *ARID1A* (**Figure 1F**). *TP53* was the most common gene to produce candidate neoantigens (n=55), which accounted for only 0.87% of all predicted neoantigens. Neoantigens predicted to be presented by human leukocyte antigen (HLA)-A molecules were the most common (48.2%; **Figure 1G**), more specifically on HLA-A*02 (20.39%; **Figure 1H**). In patients with matched RNA-seq, 32% (1,651/5,155) of predicted neoantigens were expressed, with a median of 7 per tumour sample (range 0-109; **Figure 1I**). Expressed neoantigen load was associated with tumour purity (Spearman’s r=0.32, p<0.001; **Figure 1I**).

**Figure 1:**
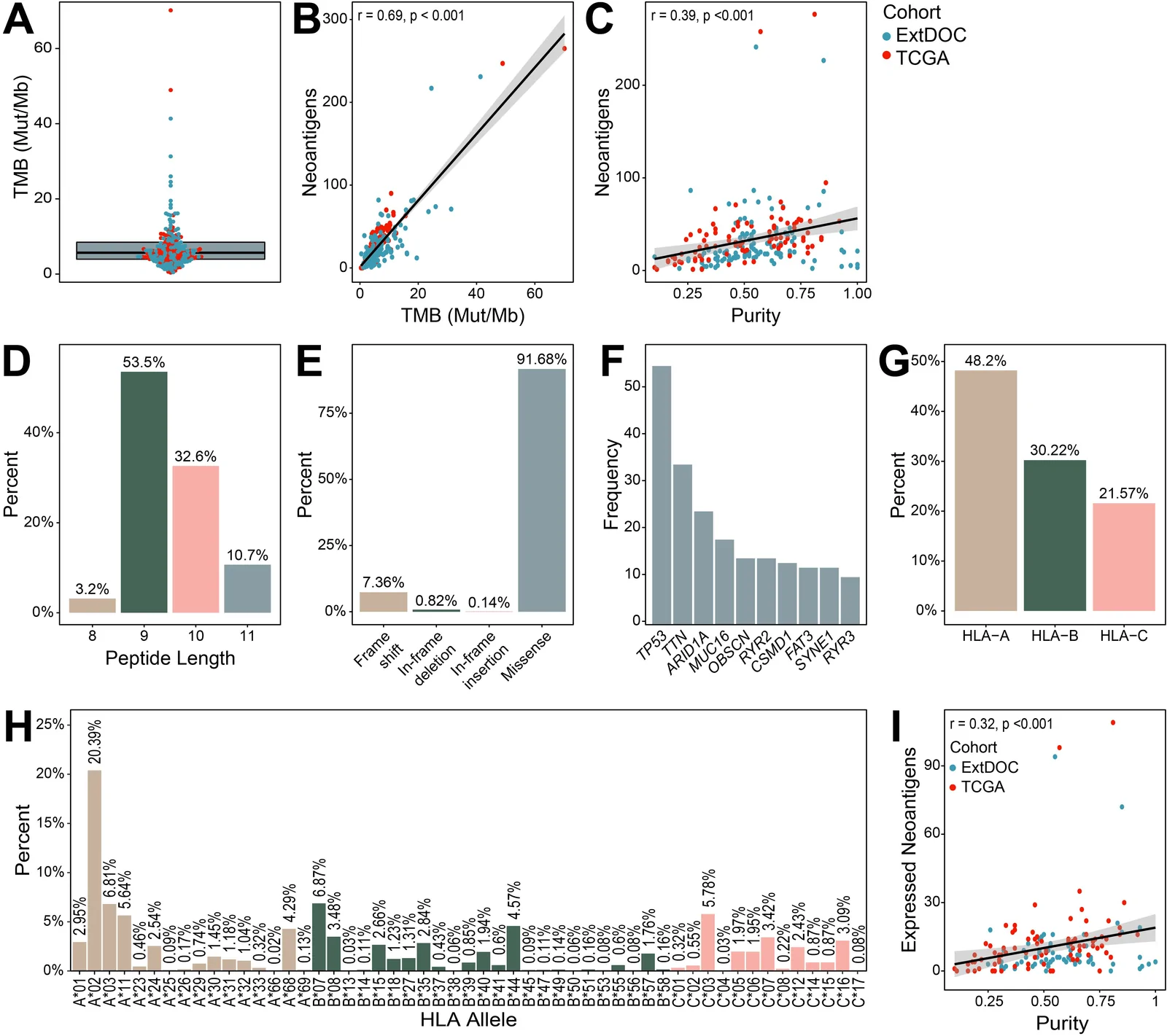
The tumour mutation, putative neoantigen, and HLA landscape in OAC tumours. **(A)** Median TMB was 5.81 (range 0.38-70.16). Correlations were found between **(B)** TMB and total putative neoantigen load and **(C)** tumour purity and total neoantigen load. **(D)** The majority of predicted neoantigens were nine amino acids in length (53.5%) and **(E)** derived from missense mutations (91.68%). **(F)** Predicted neoantigens most frequently arose from commonly mutated genes in OAC. **(G)** Most neoantigens were predicted to bind to HLA-A alleles (48.2%). **(H)** The largest proportion of neoantigens were predicted to be presented by HLA-A*02 (20.39%). **(I)** Expressed neoantigen load is correlated with tumour purity (Spearman’s r =0.32, p<0.001). TMB (Mut/Mb), tumour mutation burden (Mutations/Megabase); ExtDOC, Extended-DOCTOR; TCGA, The Cancer Genome Atlas; HLA, human leukocyte antigen.

24/6,346 (0.38%) putative neoantigens were shared between patients, with only two (SSCMGGMN**Q**R, SSCMGGMN**W**R) shared between more than two patients (**Supplementary Table 3**). Both mutant peptides originated from the same wild-type peptide sequence (SSCMGGMNRR) in *TP53*, and patients did not share the same HLA class I molecule.

We calculated the mean diffuse agretopicity index (DAI) for NAT-treated patients (n=100) to assess immunogenicity. Median DAI was 2201.57 (range 1.16-11,629.71), with a weak correlation between DAI or expressed neoantigen DAI (expDAI) and neoantigen load (r=-0.25, p<0.05; **Supplementary Figure S3A** Patients with a higher than median expDAI showed significantly better survival (median OS 46 months, vs. 24 months, p<0.05; **Supplementary Figure S3B**). This remained significant after correcting for clinical stage (p<0.05; **Supplementary Figure S3C**). No other significant associations between putative neoantigen load or DAI and clinicopathological features were observed.

### 3.3 The pre-treatment OAC immune microenvironment is associated with survival

To characterise the TME, we analysed RNA-seq data from the ExtDOC (n=115) and TCGA (n=82) cohorts. Given the cohorts displayed significantly different patient survival, we performed unsupervised clustering on inferred cell type enrichment for each cohort individually. This revealed three distinct immune clusters with significantly different TME landscapes (**Figure 2A**; **Supplementary Figure S4A**). Principal component analysis (PCA) of the ExtDOC GSVA immune cell scores showed separation into the three clusters, reflecting their distinct biology (**Supplementary Figure S4B**). No difference in tumour purity was seen between clusters (**Supplementary Figure S4C**).

**Figure 2:**
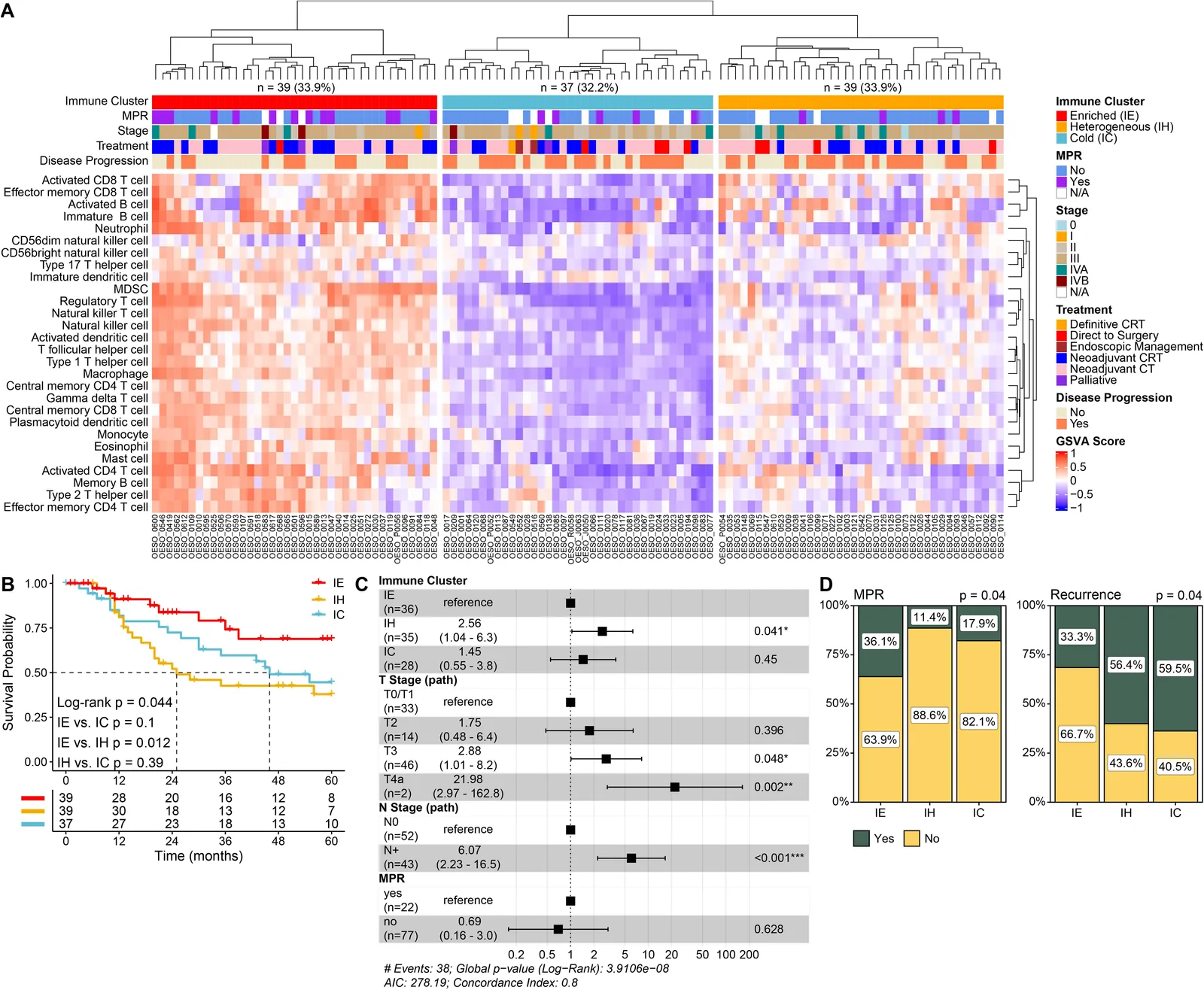
Characterisation of the immune microenvironment in OAC. **(A)** Unsupervised clustering of GSVA scores of 28 immune cell types for the ExtDOC cohort. Each row represents an immune cell type, and each column an individual patient. Clustering was performed using Euclidean distance and Ward linkage. Relevant clinicopathological annotations are included in the top annotation bars and include MPR, clinical stage, treatment, and disease progression. **(B)** Survival differences shown in a Kaplan-Meier plot for the Extended-DOCTOR patients stratified by cluster assignment. **(C)** Forest plot showing the multivariable HR models of OS vs cluster and other predictive clinicopathological characteristics in NAT-treated patients. **(D)** Clinicopathological features major pathologic response (MPR) and recurrence showed significantly different proportions across the immune clusters. CRT, chemoradiotherapy; CT, chemotherapy; GSVA, gene set variation analysis; IC, immune-cold; IE, immune-enriched; IH, immune-heterogenous; MDSC myeloid-derived suppressor cell; MPR, major pathologic response; N/A, not applicable; NAT, neoadjuvant treatment. p-value signifcance levels are indicated as * <0.05; ** <0.01; *** <0.001.

The clusters were classified as immune-enriched (IE): high immune cell infiltration; immune-heterogeneous (IH): varying degrees of adaptive and innate immune cell infiltrate; and immune-cold (IC): largely devoid of immune cell infiltration. Across both cohorts, IE patients demonstrated the best survival outcomes and IH patients the worst, despite evidence of immune infiltration (**Figure 2B**; **Supplementary Figure S4D**). Immune cluster, pathological T and N stage, and MPR were significant prognostic factors for OS in NAT-treated ExtDOC patients (n=99; **Supplementary Table 4**). Multivariate analysis confirmed the prognostic significance of immune cluster along with T and N stage (**Figure 2C**; **Supplementary Table 4**). Furthermore, significant differences in rates of MPR and disease recurrence were identified favouring IE patients (p<0.05; **Figure 2D**).

In NAT-treated ExtDOC patients, no significant differences for TMB or neoantigen load were seen between immune clusters (**Supplementary Figures S5A-C**). However, IE patients had a significantly higher expDAI than IH patients (p<0.05; **Supplementary Figure S5D**).

### 3.4 High tumour stroma is associated with worse survival

We assessed tumour immune dysfunction and exclusion (TIDE) scores to determine how stromal components influence immune infiltration and patient survival in NAT-treated ExtDOC patients (n=99). When stratified by immune cluster, the IE cluster had the highest stromal scores (**Figure 3A**). The IH cluster showed the highest TIDE exclusion scores, indicating that the poor outcomes in this cluster may be related to immune exclusion.

**Figure 3:**
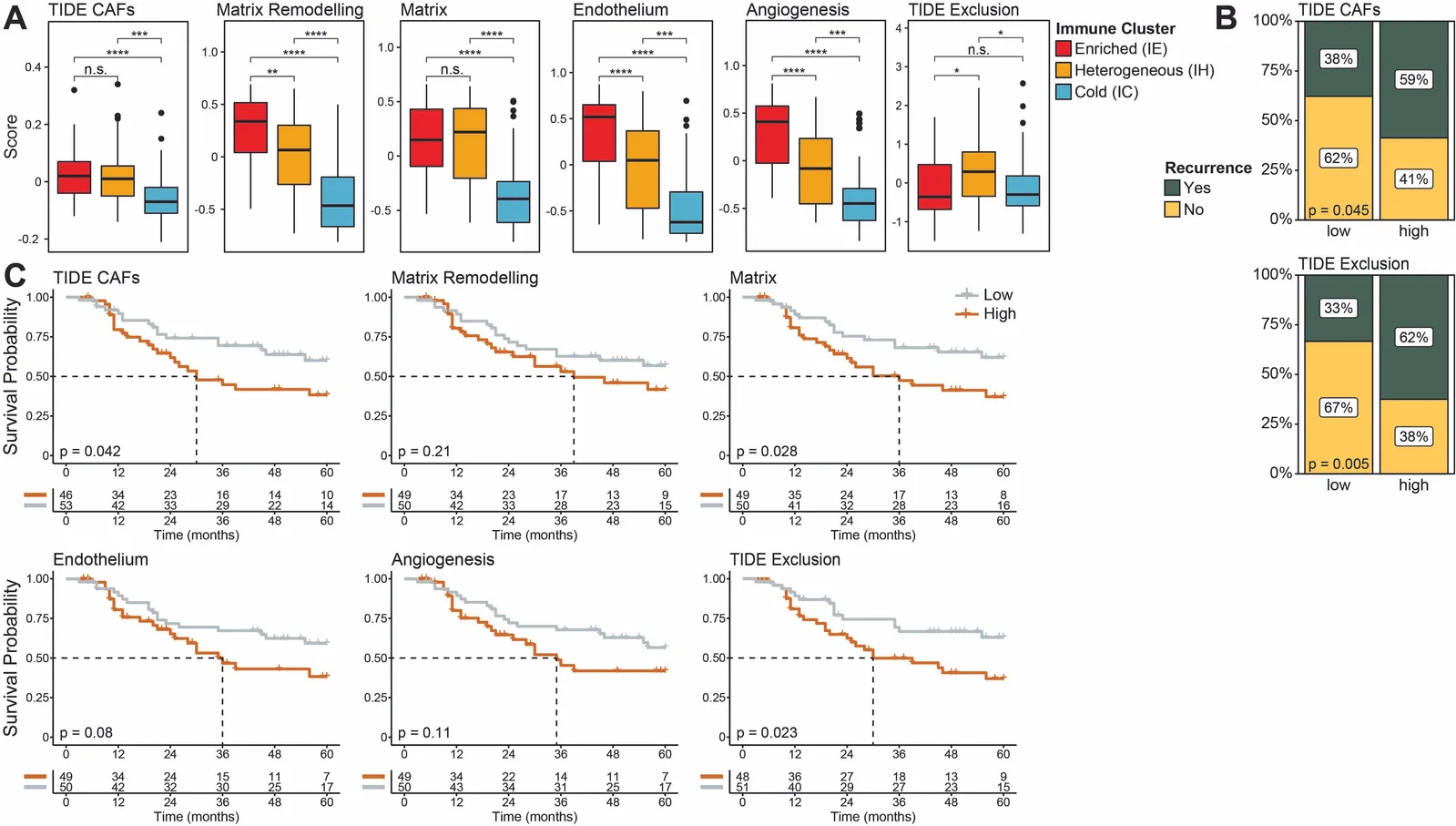
Comparison of stromal and tumour biology signatures between immune clusters. GSVA (matrix remodelling, matrix, endothelium, angiogenesis) and TIDE (CAFs, Exclusion) scores were estimated from RNA-seq of NAT-treated patients to calculate signatures. **(A)** Values per immune cluster are shown as boxplots (centre bar = median) and compared using Wilcoxon rank-sum tests. P values are indicated or represented by: n.s., non-significant; *p <0.05; **p <0.01; ***p <0.001; ****p <0.0001. **(B)** Patients stratified into the high TIDE CAFs and exclusion groups showed higher proportions of disease recurrence (p<0.05; Fisher’s exact test). **(C)** Kaplan-Meier plots comparing patients grouped by high/low scores where patients with high stromal signature scores had significantly worse overall survival. CAFs, cancer-associated fibroblasts; IC, immune-cold; IE, immune-enriched; IH, immune-heterogeneous; TIDE, tumour immune dysfunction and exclusion.

Patients with high TIDE cancer-associated fibroblasts (CAFs) and exclusion scores developed significantly more recurrences (p<0.05; **Figure 3B**). The presence of high stromal signature scores was associated with significantly worse OS (**Figure 3C**; **Supplementary Table 5**).

### 3.5 Neoantigen and immune landscape heterogeneity in OAC

We analysed two spatially separated treatment-naïve samples for 33 ExtDOC patients (**Supplementary Table 6**). Multi-region WGS data was available for 29 patients and 15 patients had matching RNA-seq data. A total of 1,916 neoantigens was identified (median 24.5, range 7-231), of which 175 were expressed (median 5, range 1-16). The median DAI was 3,073.2 (range 335.4-5,138.7), and median expDAI was 1,596.19 (range 20.02-9,817.55). We observed similar intra-tumoural TMB but differences in total neoantigen load, expressed neoantigen load, DAI, and expDAI between the multi-region samples for most patients (**Figures 4A, B**; **Supplementary Figure S6**).

**Figure 4:**
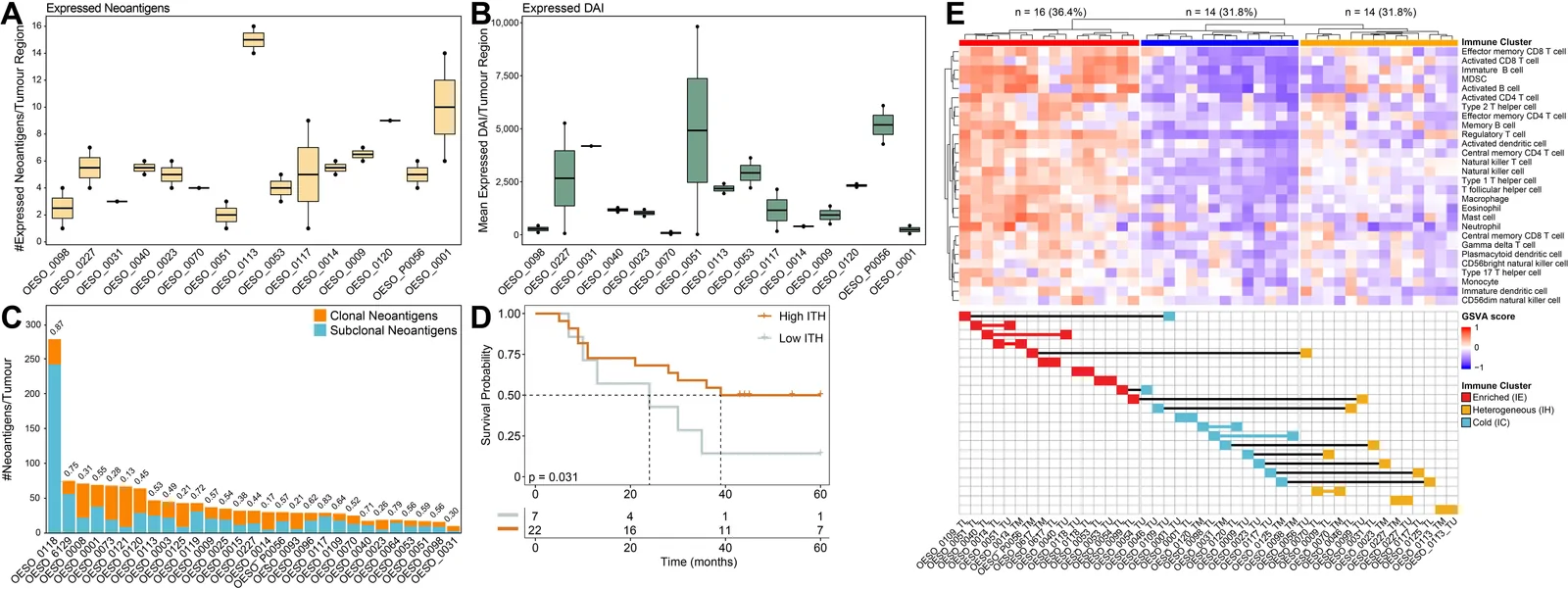
Neoantigen and immune intra-tumour heterogeneity. Comparison of **(A)** expressed neoantigen load and **(B)** expressed DAI in two spatially separated samples with matching DNA and RNA sequencing data per patient. **(C)** Clonal and subclonal neoantigen burden per patient. Clonal neoantigens were present in both tumour samples of an individual patient. **(D)** Kaplan-Meier plot for patients stratified high/low neoantigen intra-tumour heterogeneity. **(E)** Unsupervised clustering of GSVA scores of 28 immune cell types for 22 ExtDOC patients with RNA-seq data from multi-region samples. Each row represents an immune cell type, and each column an individual patient. Clustering was performed using Euclidean distance and Ward linkage. Samples from the same patient are indicated below the heatmap by connected coloured lines. DAI, diffuse agretopicity index; IC, immune-cold; IE, immune-enriched; IH, immune-heterogeneous; ITH, intra-tumour heterogeneity.

To assess the clonality of neoantigens we considered a neoantigen as clonal if it was present in both tumour samples for an individual patient, and subclonal if present in only one of the two tumour regions (**Figure 4C**). The median subclonal neoantigen burden was 54% (range 13-87%) and patients with high intra-tumour heterogeneity (ITH; upper quartile of subclonal neoantigen fraction) showed significantly better OS (p=0.031; **Figure 4D**).

Following the same approach as for the single-region samples, we used multi-region RNA-seq data from 22 ExtDOC patients to assign each sample to one of the three distinct immune clusters (**Figure 4E**). Comparing the cluster assignments from individual patients showed homogeneous immune landscapes for 12 (e.g., both samples were in the same cluster) and heterogeneous landscapes for the remaining 10 patients (e.g., samples assigned to different clusters). Only two patients had samples assigned to IE and IC, the two opposing TME profiles.

### 3.6 *In vitro* functional validation of predicted neoantigen immunogenicity

To validate our *in silico* findings, neoantigen immunogenicity was first assessed using HLA-matched healthy donor PBMCs to identify candidate neoantigens capable of eliciting neoantigen-specific cytotoxic responses. This initial functional screen identified 24/27 (88.9%) immunogenic neoantigens, which were then evaluated for neoantigen-specific T cell responses in OAC patients (**Supplementary Tables 1**, **7**; **Supplementary Figure S7**). For 14 patients PBMCs collected post-NAT (post) were available, including two patients with additional pre-NAT PBMCs (OESO_0506, OESO_0591).

Neoantigen-specific T cell responses in patients’ blood were characterised using CD107a as a marker for cell degranulation (cytotoxicity) and IFNγ, IL-2, and TNFα for polyfunctional response assessment (**Supplementary Figure S8**; **Supplementary Table 8**). Responses to neoantigens could be seen for both patients with pre-NAT samples (**Figure 5A**). Cytokine responses were also seen in post-NAT samples (**Figure 5B**). Ten patients had at least one putative neoantigen that induced polyfunctional CD8^+^ T cell responses, while six patients had at least one that failed to show any response. Notably, the only patients who failed to show neoantigen-specific cytokine responses had stage I disease (OESO_0516, OESO_0552). OESO_P0056 had two immunogenic neoantigens predicted from two spatially separated tumour regions (**Supplementary Table 7**). Specific T cell responses were detected for YLC_MT_, but none for SVK_MT_.

**Figure 5:**
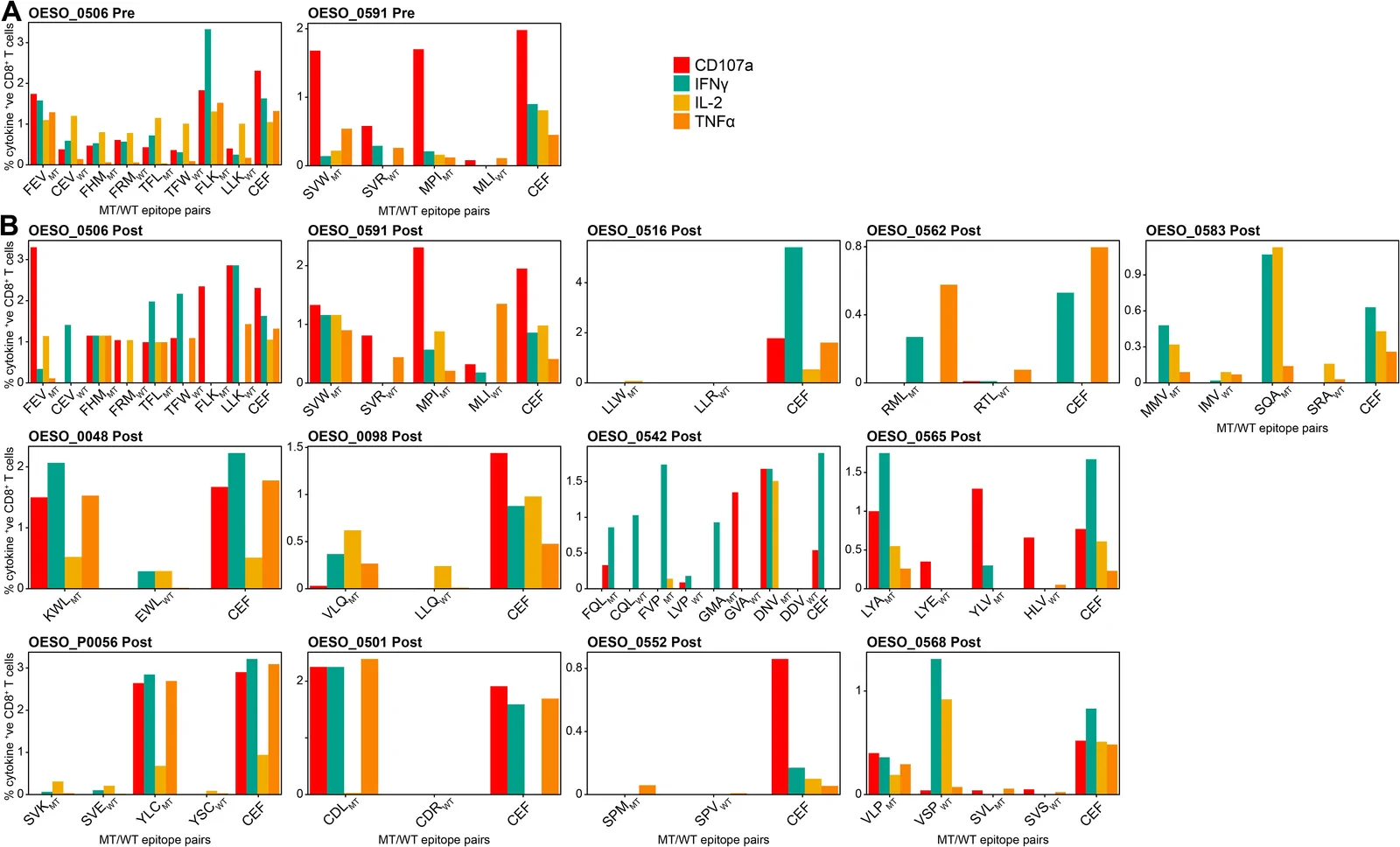
Polyfunctional cytokine responses of immunogenic tumour neoantigens. Following mock subtraction cytokine responses from patient-derived PBMCs collected **(A)** pre-neoadjuvant and **(B)** post-neoadjuvant therapy are shown. Each patient’s results along with the positive CEF control are plotted individually. MT/WT epitope pairs are adjacent to each other in each plot as indicated on the x-axis. IFNγ, interferon-gamma; IL-2, interleukin-2; MT, mutated; TNFα, tumour necrosis factor-alpha; WT, wild-type.

Comparing the pre- and post-NAT responses, all four neoantigens were able to elicit cytokine responses in pre-NAT samples, but only three showed a T cell response post-NAT for OESO_0506. For OESO_0591, only SVW_MT_ showed a specific T cell response pre-NAT, but both SVW_MT_ and MPI_MT_ were able to elicit specific T cell responses post-NAT.

We profiled the responses from CD8^+^ T cell memory subsets for neoantigens that elicited specific T cell responses in OAC patients (**Supplementary Table 9**). In pre-NAT samples, both neoantigens from OESO_0506 elicited specific T_NAIVE_ and memory cells responses. For SVW_MT_ from OESO_0591, the predominant response was from T_NAIVE_ cells. For post-NAT samples, a wide range of neoantigen-specific memory T cell responses was observed. A number of neoantigens including KWL_MT_ (OESO_0048), VLQ_MT_ (OESO_0098), and SQA_MT_ (OESO_0583) elicited specific cytokine responses from all peripheral CD8^+^ T cell subsets. Other neoantigens showed predominant cytokine responses from a single memory subset with limited responses from T_NAIVE_ cells. No patient showed responses to T_NAIVE_ cells only. Pre- and post-NAT samples from OESO_0591 showed similar CD8^+^ T cell response patterns. However, cytokine-positive responses were lower pre-NAT than post-NAT.

## 4 Discussion

Long-term survival in OAC remains limited, and improved understanding of tumour-immune interactions is needed. Here, we showed that immunogenic neoantigens and a more active TME are associated with better outcomes, and that selected neoantigens can elicit polyfunctional T cell responses in long-term survivors.

Our described mutational landscape and TMB are consistent with previous studies in OAC (7, 19, 52, 53). We observed a strong correlation between TMB and candidate neoantigen load in alignment with other studies in OAC (7, 19, 54). The general properties of neoantigen length, binding affinity, and HLA binding prediction were consistent with previous reports (55).

We found that commonly mutated genes, although more likely to produce neoantigens, accounted for a very small proportion of overall neoantigen load. *TP53*, the most commonly mutated gene in OAC, was mutated in 81% of our patients, but only produced candidate neoantigens in 26.6% of patients, and accounted for only 0.87% of all predicted neoantigens. Overall, only 0.38% of all neoantigen peptide sequences were shared between two patients, consistent with previous findings in gastrointestinal cancers (56), and the heterogeneous genomic landscape of OAC (8, 53, 57–59). Therefore, personalised neoantigen-based therapeutic strategies may enhance the efficacy of OAC treatment as observed in clinical trials for other cancers (16).

In theory, highly immunogenic neoantigens are more likely to elicit an effective immune response. We found that a high mean DAI of expressed neoantigens was associated with improved survival, which aligns with other studies showing improved response to immunotherapy and increased survival for patients with quality neoantigens (45, 60). Given this survival advantage was only observed in patients who received NAT, cytotoxic therapies may render these tumours more immunogenic (61).

We identified three immune clusters characterised by differing levels of immune infiltration reflecting distinct tumour biology within each cluster rather than a one-dimensional immune infiltration gradient. These clusters largely correlated with previous independent OAC studies (7, 34, 62). The immune-enriched (IE) tumours showed the best survival, consistent with previous OAC and pan-cancer TME studies (7, 63). NAT-treated patients with an immune-enriched TME had increased rates of MPR, and decreased rates of tumour recurrence, likely due to the predicted neoantigens in IE patients having the highest expressed DAI.

The TME is a complex milieu of tumour and immune cells as well as stromal elements including CAFs, endothelial cells, and extracellular matrix. Understanding the role of stroma in the TME is essential to improving effectiveness of therapeutic approaches. Pre-clinical models in OAC showed CAF blockade enhancing the efficacy of chemotherapy (64). Despite IE patients having a significantly higher immune cell infiltrate, no difference was seen in CAF and matrix signatures scores compared to immune-heterogeneous (IH) patients. This suggests that immune cell abundance alone may not be sufficient to confer effective anti-tumour immunity. In the IH group, higher immune exclusion scores and increased stromal infiltration may contribute to impaired immune cell function and reduced tumour control. Stromal components can influence patient outcomes through multiple mechanisms, including protecting tumour cells from chemotherapy, promoting an immunosuppressive TME, limiting immune cell trafficking, and altering immune composition (65–67). Consistent with previous findings in OAC, high stromal infiltration was associated with significantly impaired survival and increased disease recurrence (65, 68, 69). Importantly, the presence of immune infiltration within IH tumours did not translate into improved survival, suggesting that immune abundance alone is insufficient to predict effective anti-tumour responses (70). Instead, stromal-mediated immune dysfunction and immune exclusion may limit immune activity and tumour control, contributing to the poor survival observed in this group (71). For immune-cold (IC) patients, we found evidence that historical enrichment of the TME may have occurred, with mechanisms for the current immune desert phenotype being possibly driven by immunoediting (14, 72, 73). These findings suggest that different TME phenotypes may respond differently to therapeutic strategies. IE patients may benefit from neoadjuvant immune checkpoint blockade (ICB) in combination with C(R)T (e.g. phase II PERFECT trial) (74). For patients with an IH TME, anti-fibroblast therapies such as phosphodiesterase type 5 inhibition in combination with ICB and/or C(R)T may represent a promising strategy to overcome stromal-mediated immune dysfunction (64). In contrast, IC patients may require approaches to enhance anti-tumour immunity, including combinations of immunogenic cytotoxic therapies with ICB or immunotherapeutic strategies, such as neoantigen-based vaccines or adoptive cellular therapy (75). However, as these phenotypes were derived from cohort-specific clustering, prospective validation and development of reproducible classification criteria will be required before they can be used to guide clinical decision-making.

ITH remains a barrier to effective personalised therapy and genomic and immune heterogeneity have been described in OAC (8, 53, 57–59). Immunogenic neoantigens are suspected to induce immunoediting of subclonal tumour populations (36). However, knowledge of ITH with respect to the neoantigen and immune landscape is lacking. Larger differences in ITH were seen for expressed neoantigens compared to total neoantigen burden, which may reflect immunoediting due to increased immune recognition of immunogenic neoantigens (8). A high degree of neoantigen heterogeneity was associated with significantly increased OS. Heterogeneous neoantigen landscapes may promote a more effective anti-tumour immune response by increasing the repertoire of targetable neoantigens. Under this immune pressure, susceptible tumour populations are eliminated, whereas genetically distinct immune-evasive subclones may persist and expand (8, 14, 76). The neoantigen landscape is continuously reshaped through tumour evolution under immune-mediated selective pressure, resulting in ongoing immunoediting and clonal selection (77). This evolutionary process has been described in lung cancer, where immune-evasive and non-evasive subclones arise as distinct evolutionary lineages within the same tumour, highlighting the dynamic interplay between tumour evolution and anti-tumour immunity (78). Thus, timing of neoadjuvant cytotoxic therapies or immunotherapies may be important in aiding clearance of immunogenic subclones or enhancing immunogenicity of evasive tumour clones to enable immune mediated killing. Our observed intra-tumour variability in immune infiltration supports tumour evasion mechanisms such as chromosomal instability and alterations in neoantigen presentation as potential drivers of ITH in OAC (72).

Accurate selection of target neoantigens is imperative for effective neoantigen-based immunotherapies. Although computational prediction provides an efficient approach for prioritising candidate neoantigens, predicted HLA binding affinity does not necessarily translate into endogenous antigen processing, presentation, or T-cell recognition (12, 79). We have developed an *ex vivo* functional pipeline to validate the immunogenicity of *in silico* predicted putative neoantigens. Following *in silico* neoantigen prediction, we selected candidate neoepitopes for validation based on HLA class I binding, tumour mRNA expression, predicted binding affinity, and DAI (11). Cytotoxicity proved to be a highly effective functional readout for identifying immunogenic neoantigens, enabling the prioritisation of epitopes capable of eliciting potent effector T cell responses. Our *in vitro* immunogenicity testing with HLA-matched healthy donor PBMCs found most selected candidate neoantigens immunogenic, in line with findings in metastatic gastrointestinal cancers (56). Overall, our results suggest that our criteria for neoantigen selection are largely robust. As functional testing was restricted to a prioritised subset of predicted neoantigens, it is possible that additional immunogenic neoantigens were omitted from testing, including weak-binding neoantigens that have previously been shown to elicit immunogenic responses (80). Personalised cancer vaccine technology is improving and a recent Phase I/IIb clinical trial, mRNA-4157-P201/KEYNOTE-942 (17), utilised mRNA vaccine technology with room for up to 34 predicted neoantigen coding sequences. In our cohort, we identified a median of 23 predicted neoantigens per tumour sample. Although validation in larger cohorts is required, this suggests that current mRNA vaccine platforms may have sufficient capacity to target all predicted neoantigens in many patients with OAC. However, neoantigen-based therapies may need to account for ITH. In our study, only one of two region-specific neoantigens elicited specific T cell responses, suggesting multi-region sequencing could better capture putative neoantigens, maximise immune responses, and improve outcomes (13, 81).

Importantly, most immunogenic neoantigens induced polyfunctional T cell responses upon *ex vivo* re-stimulation in our OAC patients. These findings demonstrate that a subset of functionally validated neoantigens identified through our pipeline are recognised by patient-derived T cells, supporting their endogenous immunogenicity in both pre- and post-NAT PBMCs. Therefore, this assay could be used for immunomonitoring of patients.

Our pipeline, involving tumour sampling, sequencing, peptide synthesis, and immunogenicity testing, successfully identified functional neoantigens. However, our focus on patients with the HLA-A*02 allele limits generalisability to other HLA backgrounds. Furthermore, limited PBMC availability constrained technical replication and additional immune response analyses. Although functional testing confirmed immunogenicity for a subset of predicted neoantigens, additional validation is required to determine the immunogenic potential of all identified candidates. Ongoing advances in prediction algorithms and high-throughput immunoassays should improve neoantigen selection and validation (82). Despite these limitations, our study provides meaningful immune response data and establishes a foundation for future personalised neoantigen vaccine strategies.

## 5 Conclusion

This study demonstrated that neoantigen quality, tumour immune microenvironment, and intra-tumour heterogeneity contribute to the diverse clinical outcomes in patients with oesophageal adenocarcinoma. By integrating neoantigen prediction, tumour microenvironment profiling, and functional assessment of neoantigen-specific T-cell responses, we identified biologically distinct patient subgroups with different survival outcomes and patterns of immune engagement. The patient-specific nature of immunogenic neoantigens further highlights the complexity of anti-tumour immune responses while supporting personalised immunotherapies guided by individual TME and neoantigen profiles.

## Data Availability

The sequencing data presented in this study is available in the European Genome-phenome Archive, study accession number EGAS00001002864 (https://ega-archive.org/studies/EGAS00001002864). The sequencing data are generated from patient samples and therefore are available under restricted access. Data access can be granted via the EGA with completion of an institute data transfer agreement, and data will be available for a defined time period once access has been granted.
