# Supplementary material for: The neoantigen and associated tumour microenvironment in long-term survivors with oesophageal adenocarcinoma
Source: Front Immunol. 2026 Sep 21;17:1926114. doi: 10.3389/fimmu.2026.1926114 (PMC13635309; doi:10.3389/fimmu.2026.1926114)
Supplement: Supplementary file 1 [file SupplementaryFile1.docx]

Supplementary Material

# 1 Supplementary Figures and Tables

## 1.1 Supplementary Figures

Supplementary Figure S1. Overall survival comparison of the ExtDOC cohort and TCGA cohort. Kaplan-Meier survival estimates with log rank test comparing survival of ExtDOC and TCGA patients. Numbers in each group are represented in the risk table. Abbreviations: ExtDOC, Extended-DOCTOR; TCGA, The Cancer Genome Atlas.

Supplementary Figure S2. Overview of protein coding somatic mutations in OAC. (A) The oncoplot shows the top 50 genes affected by non-synonymous somatic mutations in OAC tumours from the ExtDOC and TCGA cohorts. Mutations are coloured based on their alteration type and cohort, DNA sequencing method, sex, treatment, TMB, and tumour purity are shown in the bottom colour bars. Purity values are grouped into and visualised as quartiles. (B) Tumour mutation burden and total neoantigen load for WES and WGS sequenced patients from the two independent cohorts. The Wilcoxon test was used to compare groups. Abbreviations: CRT, chemoradiotherapy; CT, chemotherapy; ExtDOC, Extended-DOCTOR; n.s., not significant; TCGA, The Cancer Genome Atlas; TMB, Tumour mutation burden; WES, whole-exome sequencing; WGS, whole-genome sequencing.

Supplementary Figure S3. DAI association with overall survival. (A) Neoantigen load was not associated with mean diffuse agretopicity index (DAI) or mean expressed DAI. (B) Kaplan-Meier survival analysis showing OS for neoadjuvant therapy treated patients stratified by mean expressed DAI. (C) Forest plot showing the multivariable Cox proportional hazards model of OS vs high/low expressed DAI and clinical stage. Abbreviations: DAI, diffuse agretopicity index.

Supplementary Figure S4. Characterisation of the immune microenvironment in OAC. (A) Unsupervised clustering of GSVA scores of 28 immune cell types for the TCGA cohort. Each row represents an immune cell type, and each column an individual patient. Clustering was performed using Euclidean distance and Ward linkage. (B) Principal component analysis revealed three distinct cohorts correlating with the unsupervised k-means clustering analysis. (C) No difference in tumour purity was observed between immune clusters. (D) Survival differences shown in a Kaplan-Meier plot for the TCGA cohort stratified by cluster assignment. Abbreviations: GSVA, gene set variation analysis; IC, immune-cold; IE, immune-enriched; IH, immune-heterogenous; MDSC myeloid-derived suppressor cell; n.s., non significant.

Supplementary Figure S5. Neoantigen profiles across the different immune clusters. (A) TMB showed no differences between the clusters. (B) Neoantigens and (C) expressed neoantigens were equally distributed across immune clusters. (D) Immune-enriched cluster showed significantly higher expressed DAI than the immune-heterogeneous cluster. Abbreviations: DAI, diffuse agretopicity index; IC, immune-cold; IE, immune-enriched; IH, immune-heterogeneous; n.s., non significant; TMB (Mut/Mb), Tumour mutation burden (Mutations/Megabase).

Supplementary Figure S6. Multi-region sample and data availability. Comparison of (A) tumour mutation burden, (B) total neoantigen burden and (C) DAI of two spatially separated samples with DNA sequencing data per patient. Abbreviations: DAI, diffuse agretopicity index; TMB (Mut/Mb), tumour mutation burden (Mutations/Megabase).

Supplementary Figure S7. Functional Validation of immunogenic tumour neoantigens. Representative plots showing the immunogenicity of putative neoantigen for two patients using HLA-matched healthy donors. These plots display specific cytotoxicity of T cells against T2A2 cells loaded with the candidate neoantigen peptides at varying effector : target ratios. FLUGIL, and a Mock condition were used as positive and negative controls, respectively.

Supplementary Figure S8. Flow cytometry assessment of CD8+ T cell functional responses to neoantigen stimulation. After 8 hours co-culture of autologous PBMCs and target neoepitopes CD8+ T cells were assessed for degranulation and polyfunctional cytokine responses to immunogenic neoepitopes and their wild-type counterparts. Responses shown correspond to the percentage of total CD8+ T cells. Representative plots for (A) a target neoepitope showing functional responses compared to the corresponding wild-type peptide and (B) a target neoepitope that failed to show functional response compared to the wild-type peptide. Abbreviations: IFNγ, interferon-gamma; IL-2, interleukin-2; MT, mutated; TNFα, tumour necrosis factor-alpha; WT, wild-type.
